# Magnetic Resonance Imaging Parameters at 1 Year Correlate With Clinical Outcomes Up to 17 Years After Autologous Chondrocyte Implantation

**DOI:** 10.1177/2325967118788280

**Published:** 2018-08-07

**Authors:** Helen S. McCarthy, Iain W. McCall, John M. Williams, Claire Mennan, Marit N. Dugard, James B. Richardson, Sally Roberts

**Affiliations:** † Robert Jones and Agnes Hunt Orthopaedic Hospital NHS Foundation Trust, Oswestry, UK.; ‡ Institute for Science and Technology in Medicine, Keele University, Keele, UK.; *Investigation performed at the Robert Jones and Agnes Hunt Orthopaedic Hospital NHS Foundation Trust, Oswestry, UK*

**Keywords:** articular cartilage, magnetic resonance imaging, histology, imaging, knee, tissue engineering

## Abstract

**Background:**

The ability to predict the long-term success of surgical treatment in orthopaedics is invaluable, particularly in clinical trials. The quality of repair tissue formed 1 year after autologous chondrocyte implantation (ACI) in the knee was analyzed and compared with clinical outcomes over time.

**Hypothesis:**

Better quality repair tissue and a better appearance on magnetic resonance imaging (MRI) 1 year after ACI lead to improved longer-term clinical outcomes.

**Study Design:**

Cohort study; Level of evidence, 3.

**Methods:**

Repair tissue quality was assessed using either MRI (11.5 ± 1.4 [n = 91] or 39.2 ± 18.5 [n = 76] months after ACI) or histology (16.3 ± 11.0 months [n = 102] after ACI). MRI scans were scored using the whole-organ magnetic resonance imaging score (WORMS) and the magnetic resonance observation of cartilage repair tissue (MOCART) score, with additional assessments of subchondral bone marrow and cysts. Histology of repair tissue was performed using the Oswestry cartilage score (OsScore) and the International Cartilage Repair Society (ICRS) II score. Clinical outcomes were assessed using the modified Lysholm score preoperatively, at the time of MRI or biopsy, and at a mean 8.4 ± 3.7 years (maximum, 17.8 years) after ACI.

**Results:**

At 12 months, the total MOCART score and some of its individual parameters correlated significantly with clinical outcomes. The degree of defect fill, overall signal intensity, and surface of repair tissue at 12 months also significantly correlated with longer-term outcomes. The presence of cysts or effusion (WORMS) significantly correlated with clinical outcomes at 12 months, while the presence of synovial cysts/bursae preoperatively or the absence of loose bodies at 12 months correlated significantly with long-term clinical outcomes. Thirty percent of repair tissue biopsies contained hyaline cartilage, 65% contained fibrocartilage, and 5% contained fibrous tissue. Despite no correlation between the histological scores and clinical outcomes at the time of biopsy, a lack of hyaline cartilage or poor basal integration was associated with increased pain; adhesions visible on MRI also correlated with significantly better histological scores.

**Conclusion:**

These results demonstrate that MRI at 12 months can predict longer-term clinical outcomes after ACI. Further investigation regarding the presence of cysts, effusion, and adhesions and their relationship with histological and clinical outcomes may yield new insights into the mechanisms of cartilage repair and potential sources of pain.

Since its inception over 20 years ago,^3^ autologous chondrocyte implantation (ACI) has been used worldwide in the treatment of chondral/osteochondral defects. Despite this, there remains a need for reliable outcome measures, particularly ones capable of predicting long-term clinical outcomes. Treatment success is often assessed with functional questionnaires such as the modified Lysholm score.^24^ Magnetic resonance imaging (MRI) is commonly utilized for the evaluation of cartilage defects and their repair, customarily using the magnetic resonance observation of cartilage repair tissue (MOCART) score.^14^ The MOCART score was specifically designed for the analysis of cartilage repair after ACI^14^ as an alternative to whole-knee scoring systems designed to assess the severity and progression of osteoarthritis, such as the whole-organ magnetic resonance imaging score (WORMS).^18^

A less common, more invasive technique for determining the quality of repair tissue is achieved by histology of a biopsy performed during “second-look” arthroscopic surgery.^10^ By necessity, this must be of a very small portion of the repair tissue, allowing the evaluation of criteria such as tissue morphology (presence/absence of hyaline cartilage) that cannot be obtained via imaging, although parameters such as lateral integration are more difficult to determine.

There have been conflicting reports in the literature as to the correlation between clinical outcomes and MRI scores,^4,6,14,22^ while others state little if any correlation between clinical outcomes and histological scores.^5,9,11,16^ Few studies, however, have assessed the relationship between histology and MRI.^19,27,28^ In addition, the scores used for clinical outcomes, MRI, and histology vary.

In this retrospective study, we examined data from patients who underwent ACI for chondral/osteochondral defects of the knee, assessed the quality of repair tissue by both imaging and histology, and compared it with clinical outcomes in the short and longer term. We hypothesized that better quality repair tissue at 12 months after ACI, resembling healthy, native articular cartilage, leads to improved midterm to long-term clinical outcomes.

## Methods

### Patients and ACI Procedure

All patients (N = 163) recruited to this study have been investigated as part of an ethically approved project (REACT 09/H1203/90, granted by the West Midlands National Research Ethics Service). Each patient underwent ACI treatment in our center for chondral/osteochondral defects in their knee using a 2-stage procedure as described previously.^3^ Macroscopically normal cartilage was harvested and processed in our on-site Good Manufacturing Practice–approved laboratory, and isolated chondrocytes were culture-expanded in monolayer for approximately 21 days. These autologous cells were then implanted during an open procedure beneath either a periosteal (ACI-P) or collagen (ACI-C) (Chondro-Gide; Geistlich Pharma) membrane. The location and approximate size of the treated defect(s) were recorded on a specifically designed knee map.^26^ Patient demographics are shown in Table 1. At approximately 12 months after ACI, patients were offered arthroscopic surgery for a repair tissue biopsy to be performed, as is common practice.

**TABLE 1:** Patient Demographic Data*^a^*

	Males (n = 118)	Females (n = 45)	Total (N = 163)
Age at the time of ACI, y	35.7 ± 9.4 (15-70)	37.2 ± 9.9 (16-65)	36.2 ± 9.5 (15-70)
Location of defect, n			
MFC	77	31	108
LFC	20	11	31
Patella	8	5	13
Trochlea	17	5	22
MTP	6	2	8
LTP	4	2	6
Total	132	56	188
Size of defect, cm^2^			
MFC	5.4 ± 3.8 (0.3-22.5)	4.1 ± 4.3 (0.5-21.0)	5.0 ± 4.0 (0.3-22.5)
LFC	4.9 ± 3.2 (1.0-12.0)	5.3 ± 2.7 (2.0-10.8)	5.1 ± 2.9 (1.0-12.0)
Patella	4.4 ± 2.9 (0.5-9.6)	1.8 ± 0.9 (1.0-3.0)	3.5 ± 2.7 (0.5-9.6)
Trochlea	5.1 ± 3.5 (0.5-12.0)	2.8 ± 3.2 (0.5-7.5)	4.6 ± 3.5 (0.5-12.0)
MTP	2.7 ± 1.3 (1.2-4.0)	5.2 ± 3.3 (2.8-7.5)	3.4 ± 2.1 (1.2-7.5)
LTP	4.9 ± 4.9 (0.5-12.0)	2.9 ± 2.8 (0.9-5.0)	4.3 ± 4.2 (0.5-12.0)
Total	5.1 ± 3.6 (0.3-22.5)	4.1 ± 3.7 (0.5-21.0)	4.6 ± 3.5 (0.3-22.5)
Patch type,*^b^* No. of defects treated			
Chondro-Gide	51	25	76
Periosteum	77	30	107

*^a^*Values are shown as mean ± SD (range) unless otherwise indicated. ACI, autologous chondrocyte implantation; LFC, lateral femoral condyle; LTP, lateral tibial plateau; MFC, medial femoral condyle; MTP, medial tibial plateau.

*^b^*For 5 treated defects, the patch type was unknown.

### Clinical Outcomes

Patient-reported modified Lysholm scores,^24^ as a measure of knee function, were obtained at baseline, at the time of biopsy and/or MRI, at yearly intervals after ACI, and at the patients’ final clinical follow-up at a mean of 8.4 ± 3.7 years (range, 2.0-17.8 years). Only those biopsies and MRI scans with a corresponding Lysholm score (completed within 4 months of biopsy or MRI) were used to analyze any correlation with clinical outcomes.

### Magnetic Resonance Imaging

A total of 241 MRI scans from 136 patients (98 male, 38 female) with a corresponding Lysholm score were included in this study. MRI was performed at a mean 1.7 ± 2.1 months before stage 1 ACI (“baseline”; range, 0-10 months; n = 74), at a mean 11.5 ± 1.4 months after ACI (“12 months”; range, 10-16 months; n = 91), and at a final radiological follow-up of 3.3 years after ACI (mean, 39.2 ± 18.5 months; range, 16-119 months; n = 76). MRI between 1998 and 2010 was performed on a 1.5-T scanner (Siemens) with (1) a T1 sagittal and coronal spin echo sequence, (2) a sagittal proton density with fat saturation (PD-FS) sequence, (3) an axial dual echo with PD-FS and T2 with fat saturation sequence, (4) a coronal short TI inversion recovery sequence, and (5) three 3-dimensional (3D) sequences: 3D Genzyme, T1 3D fast low-angle shot (FLASH) water excitation, and 3D FLASH 30° flip angle. MRI from December 2011 onward was performed on a 3-T scanner with (1) a T1 sagittal spin echo sequence, (2) a sagittal PD-FS sequence, (3) a coronal and axial PD-FS sequence, (4) a T2-star sagittal sequence, and (5) the same 3D sequences as described above. The 3D images were acquired in the sagittal plane except for patients with patellar grafts, who were viewed in the axial plane.

MRI scans were scored in a blinded fashion by an experienced orthopaedic radiologist consultant (I.W.M.) specializing in cartilage repair, with both the WORMS^18^ (score of 0 [best] to 326 [worst] at baseline, 12 months, and follow-up) and MOCART score^14^ (score of 0 [worst] to 100 [best] at 12 months and follow-up). The single subchondral bone parameter of the MOCART score, which encompasses edema, granulation tissue, cysts, and sclerosis, was expanded to record the presence of subchondral cysts (absent, 0; small, 1; large, 2; multiple, 3) and edema (absent, 0; mild, 1; moderate, 2; severe, 3) as individual parameters in addition to the single MOCART parameter and was assessed separately. The number of osteophytes (in addition to the WORMS size parameter) was also recorded.

### Repair Tissue Biopsy

A total of 102 core biopsies (1.8-mm diameter) of repair tissue formed at the site of the treated defect (using knee maps as a guidance)^26^ were performed in 81 patients (82 procedures; 61 male and 20 female). These were performed arthroscopically using a juvenile bone marrow biopsy needle (at a mean of 16.3 ± 11.0 months [range, 4-80 months] after ACI), snap-frozen in liquid nitrogen–cooled hexane, and stored at –196°C until cryosectioning. Cryosections 7 μm thick were collected onto poly-L-lysine–coated slides and stained with either hematoxylin and eosin or toluidine blue to assess the general morphology and proteoglycan content of repair tissue, respectively,^20^ and polarized light to assess collagen fiber organization and orientation. Semiquantitative scoring was performed using both the Oswestry cartilage score (OsScore; a nominal score of 0-10 with 7 parameters)^19^ and the International Cartilage Repair Society (ICRS) II score (a visual analog scale of 0-10 for each of the 14 parameters)^13^; for both scoring systems, a higher score represents better quality of repair tissue. Only those repair tissue biopsies with an MRI scan taken within 4 months of the biopsy were used to compare histology and MRI.

### Statistical Analysis

Data were tested for normality using the Shapiro-Wilk test, and subsequent analyses were performed as appropriate. Nonparametric unpaired data were analyzed for statistical differences using either the Mann-Whitney *U* test or Kruskal-Wallis test for variance (applying the Bonferroni post hoc correction). Correlations were assessed using the Spearman rank correlation. Statistical differences between grouped frequency data of the Lysholm score parameters were examined using the Pearson chi-square test of independence. A *P* value of <.05 was deemed significant. Linear regression analyses were used to determine the association between MRI scores and clinical outcomes over time. Statistical analyses were performed using Analyse-it software for Microsoft Excel (v 2.30).

## Results

### MRI Versus Clinical Outcomes

The median baseline Lysholm score was 54 (range, 21-83) and at 12 months had significantly improved to 71 (range, 21-100) (*P* < .0001). At a mean final clinical follow-up of 8.4 ± 3.7 years, the median Lysholm score had significantly dropped to 58 (range, 17-100) (*P* < .012) but remained significantly higher than at baseline (*P* < .035). Patient age at ACI did not significantly correlate with clinical outcome.

**Figure 1.**
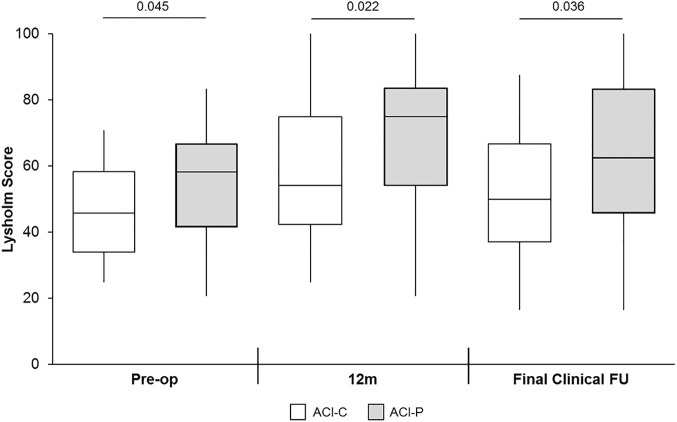
Patients treated with collagen autologous chondrocyte implantation (ACI-C) had significantly lower Lysholm scores than patients treated with periosteal autologous chondrocyte implantation (ACI-P) at each of the 3 time points. The box and the horizontal line represent the interquartile range and the median, respectively, and the whiskers represent the range. FU, follow-up.

Despite no significant difference in the size of ACI-C– and ACI-P–treated defects, patients undergoing ACI-C had significantly lower Lysholm scores preoperatively, at 12 months, and at final clinical follow-up (Figure 1). The mean follow-up for ACI-C–treated patients was significantly shorter than for ACI-P–treated patients (6.1 ± 2.7 vs 9.8 ± 3.7 years, respectively).

### MOCART Score

At 12 months after ACI, the median MOCART score was 70 (range, 0-95) and was significantly decreased at final radiological follow-up to a median of 60 (range, 0-100) (*P* = .045). It correlated significantly with the Lysholm score at both 12 months (*r* = 0.32, *P* = .0025) and at radiological follow-up (*r* = 0.35, *P* = .0032). Furthermore, 4 of the 9 scoring parameters (degree of defect fill, surface and structure of repair tissue, overall signal intensity) in addition to 1 of the 2 extra parameters scored (subchondral cysts) had a significant association with clinical outcomes (Table 2). The use of either Chondro-Gide or periosteum did not significantly affect MOCART scores at 12 months (median MOCART score of 70 for both patches), but at final radiological follow-up, defects treated with ACI-C had a significantly lower MOCART score (median, 39 [range, 0-95]) than those treated with ACI-P (median, 65 [range, 10-100]) (*P* = .012).

**TABLE 2:** Relationship Between MOCART Scoring Parameters and Clinical Outcomes at 12 Months After ACI*^a^*

MOCART Parameter	MRI Scans, %	Lysholm Score		*P* Value
		Median (Range)	IQR	
Degree of defect fill				**.002** *^b^*
Complete	47	73 (21-100)	27	
Hypertrophic	16	75 (33-92)	40	
>50% adjacent cartilage	17	81 (46-100)	19	
<50% adjacent cartilage	8	50 (33-63)	12	
Exposed subchondral bone	12	42 (29-88)	34	
Integration to border				.064*^b^*
Complete	60	75 (25-100)	27	
Incomplete	15	71 (35-100)	38	
Defect <50% repair length	12	58 (21-96)	38	
Defect >50% repair length	13	42 (29-88)	45	
Surface of repair tissue				**.002** *^b^*
Intact	57	75 (25-100)	25	
Damage <50% repair depth	21.5	77 (21-100)	32	
Damage >50% repair depth	21.5	46 (29-88)	32	
Structure of repair tissue				**.033** *^c^*
Homogeneous	55	75 (21-100)	32	
Inhomogeneous	45	58 (29-100)	38	
Overall signal intensity				**.018** *^b^*
Identical to adjacent cartilage	17	71 (42-96)	26	
Slight signal alteration	60	75 (21-100)	27	
Large signal alteration	23	46 (29-92)	39	
Subchondral lamina				.744*^c^*
Intact	38	71 (21-100)	32	
Not intact	62	71 (29-100)	33	
Subchondral bone				.485*^c^*
Intact	33	75 (33-100)	33	
Not intact	67	71 (21-100)	33	
Adhesions				.428*^c^*
Absent	76	71 (21-100)	30	
Present	24	67 (33-92)	42	
Effusion				.246*^c^*
Absent	59	75 (25-100)	29	
Present	41	58 (21-100)	38	
Subchondral cysts^*d*^				**.049** *^b^*
Absent	75	75 (21-100)	33	
Small	20	63 (29-92)	35	
Large/multiple	4	40 (33-63)	19	
Subchondral marrow edema^*d*^				.655*^b^*
Absent	37	73 (33-100)	34	
Mild	46	71 (21-96)	33	
Moderate/severe	17	60 (29-100)	42	

*^a^*Bolded *P* values indicate statistically significant association between MOCART parameter and Lysholm score. ACI, autologous chondrocyte implantation; IQR, interquartile range; MOCART, magnetic resonance observation of cartilage repair tissue; MRI, magnetic resonance imaging.

*^b^*Kruskal-Wallis 1-way analysis of variance (with post hoc Bonferroni) of MOCART parameter versus Lysholm score.

*^c^*Mann-Whitney *U* test of MOCART parameter versus Lysholm score.

*^d^*Added to standard MOCART parameters.

### WORMS Value

The median WORMS value at baseline was 18 (range, 0-74), with no significant difference at 12 months (median, 17 [range, 1-95.5]). By final radiological follow-up, the median WORMS value had risen to 28.5 (range, 3-215), which was significantly higher (ie, worse) than at both baseline (*P* = .04) and 12 months (*P* = .05). There was a significant correlation with clinical outcomes observed at 12 months (*r* = –0.21, *P* = .043) but not at baseline or radiological follow-up. There was also a significant correlation between the WORMS and MOCART score (*r* = –0.46, *P* < .0001). No significant difference in WORMS values was observed between the 2 patch types at either baseline or 12 months, but patients treated with ACI-C had significantly higher WORMS values at final radiological follow-up (median, 58.5 [range, 3-215]) (*P* = .015) than patients treated with ACI-P (median, 21.2 [range, 3-73]).

**Figure 2.**
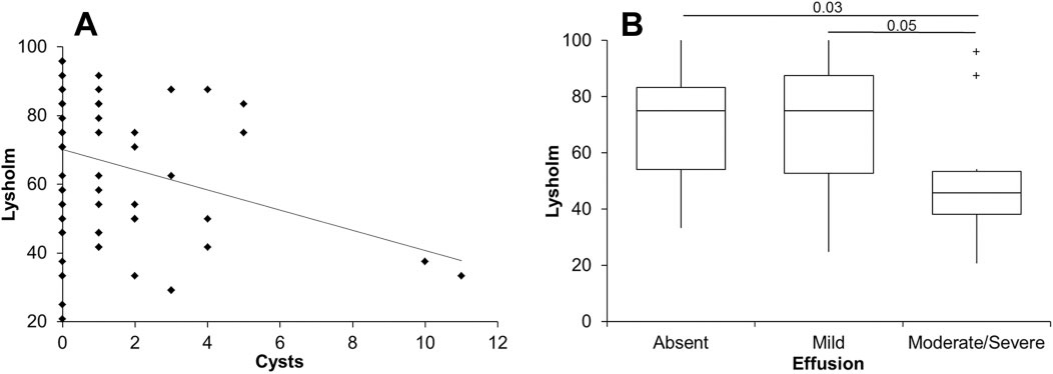
Whole-organ magnetic resonance imaging score (WORMS) values for (A) subchondral cysts and (B) effusion demonstrated a significant relationship with clinical outcomes at 12 months after autologous chondrocyte implantation. The box and the horizontal line represent the interquartile range (IQR) and the median, respectively, and the whiskers represent the range. ^+^ Outliers >1 and <3 IQR.

There was a weak but significant correlation with clinical outcomes at 12 months for the WORMS parameter of subchondral cysts (*r* = –0.25, *P* = .05) (Figure 2A). Additionally, patients with moderate/severe effusion had a significantly lower Lysholm score than patients with either mild effusion or none at all (Figure 2B). No other WORMS parameter or the number of osteophytes demonstrated any significant correlation with clinical outcomes at 12 months after ACI.

To assess if MRI could predict long-term clinical outcomes, WORMS and MOCART scores at 12 months were compared with long-term clinical follow-up Lysholm scores. While the total MOCART score at 12 months demonstrated a positive correlation with long-term clinical follow-up scores, it was not significant (*r* = 0.20, *P* = .13). However, individual parameters of the degree of defect fill (*P* = .02), surface of repair tissue (*P* = .02), and overall signal intensity (*P* = .04) at 12 months all demonstrated a positive significant relationship with final clinical follow-up scores.

For the WORMS, the presence of synovial cysts/bursae at baseline was related to significantly better clinical outcomes at follow-up than in their absence (*P* = .007). The absence of loose bodies at 12 months (*r* = 0.20, *P* = .04) was also associated with significantly better clinical outcomes at follow-up than when present.

### Male Versus Female Sex

The age at ACI was not found to be significantly different between male and female patients. Female patients had a significantly lower median baseline Lysholm score of 36 compared with 49 in male patients (*P* = .05). At 12 months, however, both male and female patients had a significant increase in the median Lysholm score to 71 (*P* < .0001). Both sexes also had a drop in the median Lysholm score at radiological follow-up to 63 (male) and 50 (female), although this was not significant.

**Figure 3.**
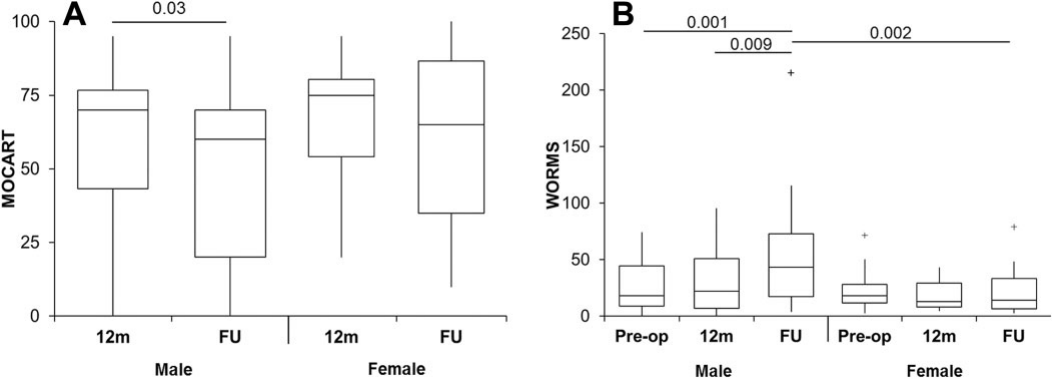
Sex-based differences in magnetic resonance imaging outcomes. (A) Magnetic resonance observation of cartilage repair tissue (MOCART) scores were significantly lower at final radiological follow-up compared with 12 months for male patients but not female patients. (B) Whole-organ magnetic resonance imaging score (WORMS) values were also significantly increased at radiological follow-up for male patients compared with baseline and 12 months but not for female patients. Male patients had significantly higher WORMS values at final radiological follow-up than female patients. The box and the horizontal line represent the interquartile range (IQR) and the median, respectively, and the whiskers represent the range. ^+^ Outliers >1 and <3 IQR. FU, follow-up.

Male but not female patients had a significant decrease in the MOCART score at radiological follow-up compared with 12 months (Figure 3A), with no significant difference in MOCART scores between the sexes at either 12 months or radiological follow-up. Male patients also demonstrated a significant negative correlation of MOCART scores over time (*r* = –0.18, *P* = .045).

The WORMS value demonstrated a significant correlation with time after ACI in male patients (*r* = 0.28, *P* = .001) but not female patients. Male patients also had a significantly higher WORMS value at final radiological follow-up than at both baseline and 12 months (Figure 3B). There was no significant difference in WORMS values in female patients between the 3 time points. Male patients had a significantly higher WORMS value than female patients at final radiological follow-up but not at baseline or 12 months. The significant correlation between WORMS and MOCART scores was not affected by sex, with male and female patients both demonstrating a significant correlation between WORMS and MOCART scores (*r* = –0.46, *P* < .0001 and *r* = –0.44, *P* = .01, respectively).

### Histology Versus Clinical Outcomes

Of the 102 biopsies analyzed, the mean OsScore value was 6.3 ± 1.6 (range, 1.3-9.5), and the mean total ICRS II score was 87.4 ± 16.5 (range, 36.7-126.6), with no significant difference between male and female patients for either scoring system. Despite only 11.8% of biopsies being predominantly hyaline cartilage, the majority of biopsies demonstrated good or excellent matrix metachromasia, surface architecture, and basal integration, with few observations of ectopic calcification or vascularization (Table 3). ICRS II scores also demonstrated a high degree of variability across all parameters in the biopsies analyzed (Table 4). The median ICRS II tissue morphology score was 5.7 (0 being fibrous tissue, 10 being perfect hyaline cartilage), reflecting the high proportion of fibrocartilage biopsies, also demonstrated by the OsScore. Additionally, the median score for basal integration was 8.7 (0 as worst, 10 as best), also reflecting the OsScore. The median score for tidemark formation was poor at 2.1, but scores ranged from 0.0 to 9.8, again reflecting the heterogeneous nature of the repair tissue biopsies.

**TABLE 3:** Association of Clinical Outcomes With OsScore Scoring Parameters at 12 Months After ACI*^a^*

OsScore Parameter	Biopsies, %	Median Lysholm Score	*P* Value
Tissue morphology			.479*^b^*
Hyaline	11.8	63	
Hyaline/fibrocartilage	18.6	53	
Fibrocartilage	64.7	74	
Fibrous tissue	4.9	62	
Matrix metachromasia			.615*^b^*
Normal	46.0	74	
Moderate	41.0	68	
Abnormal	13.0	62	
Clusters			.707*^b^*
None	54.9	69	
≤25% total cell number	36.3	70	
>25% total cell number	8.8	53	
Surface			.335*^b^*
Near normal	19.0	62	
Moderately irregular	42.9	63	
Irregular	38.1	75	
Basal integration			.314*^b^*
Good	63.6	65	
Moderately irregular	31.8	75	
Poor	4.5	39	
Calcification			.605*^c^*
Absent	68.6	63	
Present	31.4	75	
Vascularization			.102*^c^*
Absent	93.1	70	
Present	6.9	40	
Total	N/A	N/A	.745*^d^*

*^a^*ACI, autologous chondrocyte implantation; N/A, not applicable; OsScore, Oswestry cartilage score.

*^b^*Kruskal-Wallis 1-way analysis of variance (with post hoc Bonferroni) of OsScore parameter versus Lysholm score.

*^c^*Mann-Whitney *U* test of OsScore parameter versus Lysholm score.

*^d^P* value after Spearman rank correlation of total OsScore value versus Lysholm score.

**TABLE 4:** Association of Clinical Outcomes With ICRS II Scoring Parameters at 12 Months After ACI*^a^*

	ICRS II Score		
ICRS II Parameter	Median (Range)	IQR	*P* *^b^*
Tissue morphology	5.7 (0.7-9.8)	1.7	.427
Matrix metachromasia	7.0 (0.6-9.9)	3.3	.357
Cell morphology	4.4 (0.0-9.9)	5.1	.999
Chondrocyte clusters	9.9 (0.2-10.0)	1.9	.543
Surface architecture	5.5 (0.5-10.0)	4.1	.377
Basal integration	8.7 (0.6-10.0)	3.3	.809
Tidemarks	2.1 (0.0-9.8)	3.8	.749
Subchondral bone abnormalities	7.8 (1.5-9.9)	2.2	.228
Inflammation	10.0 (8.1-10.0)	0.0	.224
Calcification	10.0 (0.4-10.0)	1.5	.590
Vascularization	10.0 (0.7-10.0)	0.0	.103
Surface/superficial assessment	4.9 (0.3-9.3)	2.5	.491
Middle/deep assessment	5.3 (0.6-9.6)	2.2	.851
Overall assessment	4.9 (0.6-9.5)	2.5	.835

*^a^*ACI, autologous chondrocyte implantation; ICRS, International Cartilage Repair Society; IQR, interquartile range.

*^b^P* value after Spearman rank correlation of ICRS II parameter versus clinical outcome.

When comparing the OsScore parameters with Lysholm scores, no patients whose biopsies consisted solely of hyaline cartilage complained of constant pain. In contrast, all patients whose biopsies were fibrous tissue complained of some degree of pain, and 50% of patients complaining of constant pain had poor basal integration. All patients with vascularization present in repair tissue complained of some degree of pain, while all patients with fibrous tissue or vascularization had some degree of limp. There was not, however, any significant correlation or association with any of the histological parameters assessed by either scoring system in relation to clinical outcomes at 12 months (Tables 3 and 4) or at final clinical follow-up (mean, 8.5 ± 3.6 years [range, 2.1-16.4 years]).

### MRI Versus Histology

There were 54 patients (55 procedures) who underwent both a biopsy and an MRI within 4 months of each other, resulting in a total of 59 biopsies and 56 MRI scans. For this subgroup, the mean time of biopsy was 13.3 ± 5.6 months (range, 8-40 months), and the mean time of MRI was 12.7 ± 6.6 months (range, 6-42 months). Overall, there was no significant correlation between the total OsScore or ICRS II score with the overall MOCART score or WORMS.

**Figure 4.**
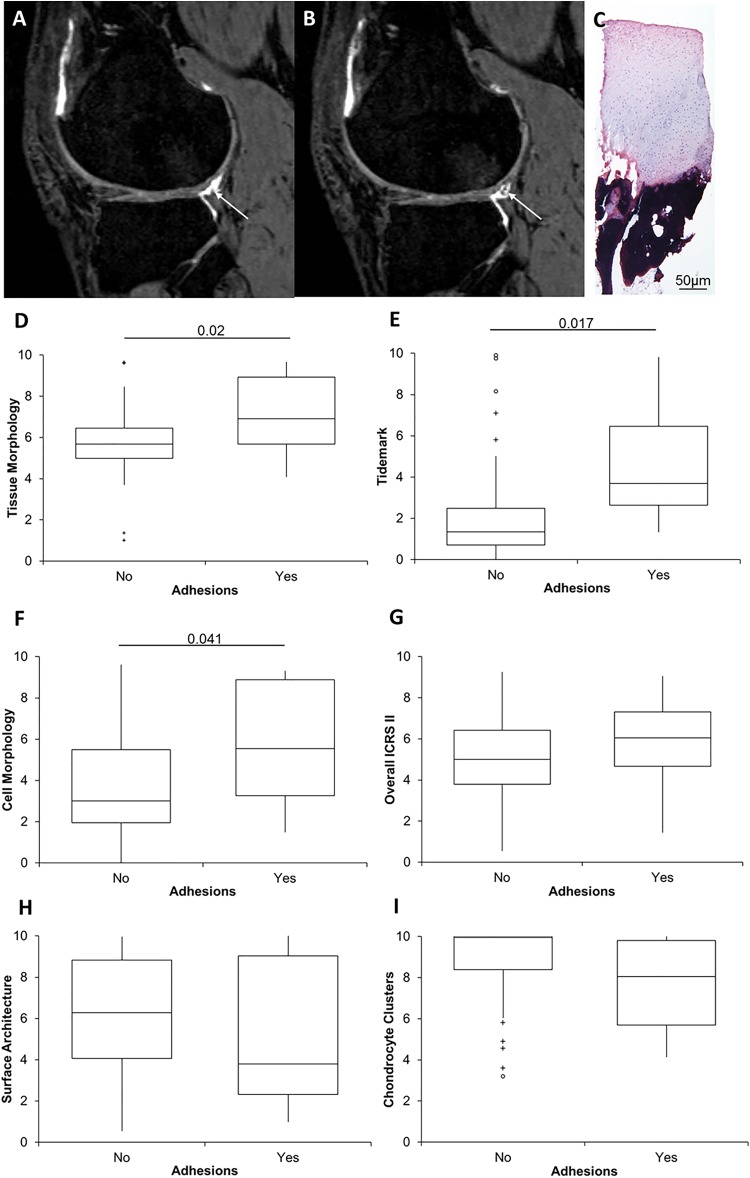
The relationship between the presence of adhesions identified by magnetic resonance imaging (MRI) and histology. (A, B) Visible adhesions observed on consecutive slices of an MRI scan (white arrows) demonstrated a significant relationship with (C, representative hematoxylin and eosin–stained biopsy) repair tissue and International Cartilage Repair Society (ICRS) II histological scores for (D) tissue morphology, (E) presence of tidemarks, and (F) cell morphology. Noticeable trends were also observed for (G) the overall ICRS II score, (H) surface architecture, and (I) chondrocyte clusters. Biopsy performed on a 44-year-old male patient of the treated defect 12 months after autologous chondrocyte implantation; the corresponding MOCART and WORMS values for this patient were 5 and 54.5, respectively. The box and the horizontal line represent the interquartile range (IQR) and the median, respectively, and the whiskers represent the range. ^+^ Outliers >1 and <3 IQR.

A negative trend was observed between the ICRS II subchondral bone and tidemark parameters and the MOCART parameters of degree of defect fill and signal intensity, respectively. There was also a significant negative correlation between the number of osteophytes present in the joint and the tidemark score of repair tissue (*r* = –0.26, *P* = .042). Interestingly, the presence of adhesions on MRI, observed in 10 of 54 patients, correlated with significantly better tissue morphology, significantly better tidemark formation, and significantly better cell morphology (Figure 4, A–F). Although not quite reaching significance, the presence of adhesions also correlated with a higher overall ICRS II score but worse surface architecture and more chondrocyte clusters (Figure 4, G–I). Furthermore, those patients with adhesions present had a significantly lower overall MOCART score (median, 20 [range, 0-80]) (*P* = .0001) than those without (median, 75 [range, 10-95]). Likewise, the WORMS value was also significantly higher in the presence of adhesions (median, 40 [range, 4-115.5]) (*P* = .015) compared with no adhesions (median, 14 [range, 3-105.5]). Neither the structure of repair tissue (on MRI) nor the overall signal intensity had any correlation with the tissue morphology of the biopsies assessed histologically.

The presence of hyaline cartilage to any extent in repair tissue did not show a significant relationship with the overall MOCART score compared with repair biopsies containing only fibrocartilage. Only 2 biopsies in this subgroup (ie, with an MRI scan at a similar time point) were categorized as fibrous tissue, and these had an overall MOCART score of 80 and 25. Neither of these biopsies had poor basal integration. The overall MOCART score did not significantly correlate with either the presence or severity of ectopic calcification or vascularization, as seen histologically.

## Discussion

The ability to detect, measure, and assess the success of cartilage repair in a minimally invasive manner has great appeal to both the clinician and particularly the patient. If this can be performed at a relatively early stage and can predict the likely long-term success, it will be beneficial for clinical trials, especially in slowly developing conditions such as osteoarthritis after a cartilage injury. Imaging modalities such as MRI have considerable capability, but despite having been used as a diagnostic tool for many years, its accuracy and value in assessing clinical outcomes in patients after treatment have remained under scrutiny. Meta-analyses and systematic reviews have demonstrated wide variability in the choice of clinical outcome measures used for assessing the relationship between MRI and clinical outcomes.^2,4^ The majority of studies have focused solely on the region of repair cartilage and not the whole joint. In this study, we have utilized 2 different MRI scoring systems to determine the impact and efficacy of autologous cell therapy in relation to both clinical outcomes and histology. To that effect, we have demonstrated the ability of MRI at 12 months after ACI to predict clinical outcomes at a mean of 8.4 years after treatment. This current study enhances our previous findings^19,27^ with the inclusion of greater numbers of patients, their repair tissue biopsies and MRI scans, the addition of an analysis of the correlation to clinical outcomes, and more in-depth histological and radiological scoring systems.

The results presented in this study demonstrate a significant positive correlation at 12 months between the MOCART score and clinical outcomes after ACI for the treatment of chondral/osteochondral defects. In addition, these results indicate that the assessment of features such as the degree of defect fill, surface of repair tissue, and overall signal intensity by MRI can be used to predict long-term clinical outcomes. Repair tissue with a smooth surface, well integrated with surrounding native cartilage, is necessary for maintaining a functional, intact joint surface. Likewise, a homogeneous structure of repair visible on MRI is indicative of healthy cartilage, with typical cartilage layers formed within repair tissue free of fissures and clefts.^15^

The MOCART score is one of the most commonly used scoring systems for the MRI assessment of cartilage repair. As with most scoring systems, the MOCART score has also been subject to “improvement”; the original score has been superseded by a modified 3D MOCART score,^29^ with the categories within the original (2-dimensional) MOCART score having been expanded on (such as degree of defect fill and effusion) or combined (such as surface and adhesions), or having new ones added altogether (such as bone interface and chondral osteophytes). As the majority of the MRI scans analyzed in this particular study were historical and stretched back almost 20 years to when more simple MRI sequences were taken, the 3D MOCART score was unfortunately not possible to use in many of our analyses. However, because the MOCART score is restricted to assessing repair cartilage only and the patients’ clinical well-being is likely be affected by more than just this, we also used the WORMS to assess the status of the whole joint.

The WORMS was developed as a multifeature evaluation of the knee, designed for use in osteoarthritis.^18^ Although cumbersome in its analysis of 14 parameters across a total of 14 regions, the scoring system has demonstrated good interobserver agreement by both its creators and our group (unpublished data). A modified WORMS has since been developed,^25^ reducing the number of anatomic regions to only 6, by assessing areas such as the medial and lateral condyles and the tibial plateau as whole entities and not examining anterior, posterior, and central regions individually. We chose, however, to adhere to the original parameters to allow for a more in-depth examination of the joint, with the exception of the “S” region, which was not scored in this study.

Despite the fact that the WORMS and MOCART were designed for different applications, some parameters overlap between the 2 scoring systems, for which we observed similarities in the results between the scores and their correlation with clinical outcomes. For example, both scoring systems demonstrated a reduction in the Lysholm score at 12 months after ACI when there was effusion present, although this was only statistically significant for the WORMS. This may be because the WORMS categorizes the level of effusion across the whole joint rather than simply questioning its presence or absence, as in the more restrictive repair-only region assessed by the MOCART score. Both scoring systems also demonstrated a significant correlation between the presence and severity of subchondral cysts and a reduction in clinical outcomes. Taken together, this suggests that effusion and subchondral cysts are associated with pain and other debilitating symptoms, in keeping with other studies.^6,23^ Thus, future research may benefit from the additional use of questionnaires that assess pain alongside physical function in more detail, such as the Western Ontario and McMaster Universities Osteoarthritis Index (WOMAC), when assessing clinical outcomes,^1^ particularly as sources of pain within such joints are unknown. The finding of the presence of synovial cysts/bursae at baseline correlating with significantly better Lysholm scores at clinical follow-up is interesting. It is possible that the implanted cells affected these tissues also; it is likely that some of the implanted chondrocytes were progenitor cells and so could have similar properties to mesenchymal stem cells, which are known to have paracrine influences possibly including anti-inflammatory effects.

Second-look arthroscopic surgery is still considered an important procedure for assessing the success of cartilage repair techniques,^10^ although it is not undertaken in all studies. However, we found little or no significant correlation between the histological assessment of repair tissue and clinical outcomes, as previously reported by others,^5,11,16^ which raises questions about the predictive usefulness of histology. Despite this, we have demonstrated some interesting relationships between the Lysholm score parameters of pain and limping with histological parameters of repair tissue morphology, basal integration, and vascularization. While the ability to microscopically assess repair tissue is invaluable for learning more about the biology of repair processes involved, it is important to remember that a repair tissue biopsy only enables the evaluation of repair at a single point of location and time in potentially actively remodeling tissue. Therefore, pinpointing any particular histological feature(s) apparently associated with pain levels could prove challenging.

Significant correlations between histology and MRI have previously been identified for clinical indications such as prostate cancer, epilepsy, and amyotrophic lateral sclerosis.^7,12,17^ We have seen a few significant correlations between histology and MRI in our study but not many, perhaps reflecting the different and often complementary types of information provided by the 2 modalities. For example, we have previously demonstrated that the use of ACI-C results in significantly superior quality of repair tissue when assessed histologically,^16^ but such findings were not reflected in the present study when assessed by MRI; the 2 patch types were not significantly different at 12 months. The significantly inferior quality of repair tissue (as assessed by MRI) after ACI-C compared with ACI-P seen in this study, however, demonstrates the importance of longitudinal follow-up and the location- and time-dependent nature of histological assessments.

We have shown an interesting correlation between the presence of adhesions identified on MRI and significantly better histological features in repair tissue. Adhesions are common after knee surgery^8^ and are associated with knee stiffness and arthrofibrosis, and thus, their correlation with significantly better histological scores is surprising. Despite this, we did not observe a significant relationship between the presence of adhesions and clinical outcomes. Adhesions are difficult to diagnose on MRI, and the severity of adhesions rather than their presence alone may be important in detecting a significant relationship with clinical outcomes. Could these “adhesions” perhaps be processes of other tissues (such as synovium) that are actually aiding in the repair or growth of the tissue?^21^ Future studies regarding the cause of adhesions, their role in tissue regeneration, and their relationship with cartilage morphology could indicate the mechanisms of repair not yet realized.

This study has some limitations. One of these is that it is a retrospective study of routinely treated patients; data were therefore not collected as systematically or at matched time points, for example, as they might have been in a clinical trial. Additionally, there was a wide range of disease severity within the treated joints, thus making comparisons more difficult—another factor that could be better controlled in a clinical trial. A further limitation of the study could be the wide age range of patients treated, up to the age of 70 years, reflecting the type of patients who typically present to clinics in a secondary or tertiary referral orthopaedic center such as ours. We found no significant effect of age on clinical outcomes; in fact, the 2 oldest patients in the study, aged 65 and 70 years, had Lysholm scores of 92 and 100 after 8 and 7 years’ follow-up, respectively. Only 1 radiologist scored the MRI scans, which could be considered a limitation. However, this radiologist was extremely experienced, in addition to which the interclass correlations with another radiologist for the MOCART and WORMS scores were 0.55 and 0.92, respectively.

## Conclusion

These results are encouraging in showing a significant association between the MOCART score and some of its individual parameters with both short-term and longer-term clinical outcomes in ACI-treated patients. This highlights the potential for MRI at 1 year to predict long-term clinical outcomes after cartilage repair, such as may be useful in clinical trials. Whereas the MOCART score assesses the complete graft, histology only examines a small, discrete region and cannot assess parameters such as lateral integration, hypertrophy, and subchondral cysts. Combining MRI with histology allows a more complete assessment of repair tissue. Further investigation regarding the presence of cysts, effusion, and adhesions as identified on MRI and their relationship with both histological and clinical outcomes may yield new insights into the mechanisms of cartilage repair and provide information to further understand pain-generating mechanisms not only in knees with focal cartilage defects but also in osteoarthritis.
